# Information clarity about Covid-19 in Indonesia: does media exposure matter?

**DOI:** 10.1186/s12889-022-13961-9

**Published:** 2022-08-12

**Authors:** Setia Pranata, Agung Dwi Laksono, Eka Denis Machfutra, Ratna Dwi Wulandari

**Affiliations:** 1 Research Center for Public Health and Nutrition, National Research and Innovation Agency Republic of Indonesia, Jakarta, Indonesia; 2 Department of Administration and Health Policy, Faculty of Public Health, Universitas Airlangga, Universitas Airlangga Campus C Mulyorejo, Surabaya, 60115 Indonesia

**Keywords:** Covid-19, Media exposure, Big data, Online survey, Public health

## Abstract

**Background:**

Confusion of information has also colored the course of the Covid-19 pandemic in Indonesia. The study analyzes the relationship between media exposure and information clarity about Covid-19 in Indonesia.

**Methods:**

The study collected extensive data (*n* = 5,397). The study determines information clarity about Covid-19 based on respondents' admissions. There were four types of media exposure analyzed: frequency of reading a newspaper/magazine, frequency of listening to a radio, frequency of watching television, and frequency of internet use. The study carried out a binary logistic regression test in the final stage.

**Results:**

The results show read a newspaper/magazine every day is 1.670 times more likely than reading a newspaper/magazine > five days a week to get precise information about Covid-19. Reading a newspaper/magazine 2–3 days a week is 1.386 times more likely than reading a newspaper/magazine > five days a week to get precise information about Covid-19. Reading a newspaper/magazine 4–5 days a week is 1.470 times more likely than reading a newspaper/magazine > five days a week to get precise information about Covid-19. Watching television 2 to 3 days a week is 1.601 times more likely than watching television > five days a week to get clear details about Covid-19. Watching television 4 to 5 days a week are 1.452 times more likely than watching television > five days a week to get precise information about Covid-19.

**Conclusion:**

The study concluded two types of media exposure related to information clarity about Covid-19 in Indonesia: the frequency of reading newspapers and watching television.

## Introduction

World Health Organisation declared COVID-19 a global pandemic [1]. In Indonesia, the first case was found in March 2020 and significantly raised. The disease risks many morbidities and even mortalities [2]. There were 706.837 confirmed cases and 20.994 deaths from March 2020 until December 2020 [3].

The spread of COVID-19 impacts the number of deaths. In addition, the disease expands and affects both economic sectors and social structures. The willingness to pay for domestic needs is lowering, and the market's insecurity makes both micro and macro businesses discontinued. The commodity fell as well as the number of export [4, 5].

The government of the Republic of Indonesia then issued COVID-19 as a national disaster. Therefore, they make a policy to the national strategy for health quarantine counterfeit and vaccination. Vaccination is an effort to address the pandemic all around the world. By this, people will move to herd immunity. The government allocates 13.92 trillion rupiahs for this program [6]. Economically, this prevention saved the national budget rather than medications [7, 8].

Generally, COVID-19 vaccination appears controversial, either pros or cons. This issue occurs as the media share information that confuses people; information clarity is not fulfilled. Whether or not the news is clear and valid, this situation may lead to misinformation and disinformation. This situation is inseparable from the many hoaxes circulating [9–11]. Such a dire situation may have resulted from celebrities' and influencers' views on the conspiracy theory surrounding the COVID-19 pandemic [12–14]. For example, Donald Trump, the ex-president of the United States, believed that the conspiracy theory brought about the pandemic as a form of resistance to government power [15].

Moreover, some studies on the acceptance of people toward COVID-19 injection were published before clinical trial number three. ITAGI survey in collaboration with WHO and the Ministry of Health indicated that 65% of people stated that they had the willingness to get the vaccination. The rest was 8% rejected, and 27% were doubtful [16].

The epidemic will impact mental disorders. The symptoms were anxiety, irrationality, stigma, and blaming for patients, the community, and health workers [13, 17]. The disease caused correlated to non-biomedical explanations [18, 19].

Delivering information is crucial to all people in cities and villages in Indonesia. This activity requires support from stakeholders and local organizations in health. Local context determines social relationships in society, and the bonding between government and communities will impact the response to the intervention [20].

The fatigue and demotivation of people facing COVID-19 become the government's responsibility to handle. The massive circulation of information via mass media (online or offline) distracts people from accessing clear and valid ones. However, the government has to build strategies to raise the trustworthiness of people up, including vaccination issues. Motivating people to obey health protocols is a must. They wear masks, apply social distancing, wash hands with soap, or use hand sanitizer.

The government uses various media to communicate and inform health protocols and vaccination, and the main issue is whether or not the information can easily be accessed and understandable. Is there a relationship between media exposure and information clarity about COVID-19 in Indonesia? Therefore, this study analyzes the relationship between media exposure and information clarity about COVID-19 in Indonesia.

## Methods

### Data source

The study collected data through online surveys of the community living in all Indonesia regions. Meanwhile, the survey collected data for one month (April 23 to May 22, 2021). At the end of data collection, the study obtained many respondents, 5,397.

Because the data in this study were collected through an online survey, the population in this study is Indonesian residents with internet access. This situation is also a limitation of this study. However, this online survey method is the best choice during the pandemic. Online surveys can collect massive data in a short time and with minimal effort.

### Variables

The study employed information clarity about Covid-19 as the outcome variable. The study determines information clarity about Covid-19 based on respondents' admissions or perceived information clarity. Information clarity about Covid-19 consists of two categories: No and Yes.

Meanwhile, the study used media exposure as an exposure variable. The respondent's acknowledgment of exposure to newspaper/magazine, radio, television, and internet media was media exposure. The frequency of reading a newspaper/magazine, listening to a radio, watching television, and frequency of internet use consists of four types: every day, 2–3 days a week, 4–5 days a week, and > 5 days a week.

Moreover, the study employed six control variables: age, gender, religion, marital status, education level, and work type. The age group was the respondent's acknowledgment of the last birthday. The age group is divided into six categories, namely 18–24, 25–34, 35–44, 45–54, 55–64, and > 64. Gender consists of two categories, namely male and female. Religion consists of six categories: Moslem, Christian, Catholic, Hinduism, Buddhism, etc. Marital status consists of never married, married, and divorced/widowed. Education level was the respondent's recognition of the level of education passed. The education level consists of primary, secondary, and higher. The study divided work types into seven groups: no work, public servants/army/police, health workers, entrepreneurs, private sector, farmer/fisherman/labor, and other types.

### Data analysis

The study evaluated all variables at the start of the analysis to verify no collinearity between independent variables. The researchers then employed chi-square to assess variations in information clarity about Covid-19. Finally, the study used binary logistic regression for multivariable analysis. A *p*-value less than 0.05 is considered statistically significant [21]. Furthermore, the author employed the IBM SPSS 26 program for all statistical analyses.

## Results

The analysis results inform that about 85.8% of people in Indonesia claim to have received precise information about Covid-19. Meanwhile, Table 1 shows the descriptive statistics of media exposure characteristics of information clarity about Covid-19 in Indonesia. The result informs that daily reading a newspaper/magazine ruled both categories of information clarity about Covid-19 in Indonesia. People listening to the radio > 5 days a week occupied both types of information clarity about Covid-19 in Indonesia. Meanwhile, watching television every day dominated both categories of information clarity about Covid-19 in Indonesia. Moreover, people using the internet daily control both types of information clarity about Covid-19 in Indonesia.

**Table 1:** Descriptive statistic of media exposure characteristics of information clarity about Covid-19 in Indonesia (*n* = 5,397)

Media Exposure Characteristics	Information clarity about Covid-19		*p*-value
	**No** **(*n* = 765)**	**Yes** **(*n* = 4,632)**	
Frequency of reading a newspaper/magazine			* < 0.001
- Every day	363 (47.5%)	2,602 (56.2%)	
- 2–3 days a week	205 (26.8%)	1,157 (25.0%)	
- 4–5 days a week	63 (8.2%)	357 (7.7%)	
- 5 days a week	134 (17.5%)	516 (11.1%)	
Frequency of listening to the radio			0.159
- Every day	136 (17.8%)	918 (19.8%)	
- 2–3 days a week	186 (24.3%)	1,174 (25.3%)	
- 4–5 days a week	62 (8.1%)	429 (9.3%)	
- 5 days a week	381 (49.8%)	2,111 (45.6%)	
Frequency of watching television			* < 0.001
- Every day	318 (41.6%)	2,680 (57.9%)	
- 2–3 days a week	152 (19.9%)	860 (18.6%)	
- 4–5 days a week	70 (9.2%)	269 (5.8%)	
- > 5 days a week	225 (29.4%)	823 (17.8%)	
Frequency of internet use			0.371
- Everyday	707 (92.4%)	4,345 (93.8%)	
- 2–3 days a week	26 (3.4%)	135 (2.9%)	
- 4–5 days a week	7 (0.9%)	45 (1.0%)	
- > 5 days a week	25 (3.3%)	107 (2.3%)	

Note: ^∗^*p* < 0.05

Table 2 shows the descriptive statistics of demography characteristics of information clarity about Covid-19 in Indonesia. The results show age group of 25–34 ruled both categories of information clarity about Covid-19 in Indonesia. Based on gender, female respondents occupied both types of information clarity about Covid-19. Regarding religion, Muslim respondents ruled out both categories of information clarity about Covid-19.

**Table 2:** Descriptive statistic of demography characteristics of information clarity about Covid-19 in Indonesia (*n* = 5,397)

Demography characteristics	Information clarity about Covid-19		*p*-value
	**No** **(*n* = 765)**	**Yes** **(*n* = 4,632)**	
Age group			* < 0.001
- 18–24	21 (2.7%)	143 (3.1%)	
- 25–34	450 (58.8%)	1,728 (37.3%)	
- 35–44	181 (23.7%)	1,177 (25.4%)	
- 45–54	83 (10.8%)	1,139 (24.6%)	
- 55–64	25 (3.3%)	370 (8.0%)	
- > 64	5 (0.7%)	75 (1.6%)	
Gender			0.884
- Male	367 (48.0%)	2,209 (47.7%)	
- Female	398 (52.0%)	2,423 (52.3%)	
Religion			*0.012
- Moslem	653 (85.4%)	3,989 (86.1%)	
- Christian	66 (8.6%)	367 (7.9%)	
- Catholic	26 (3.4%)	157 (3.4%)	
- Hinduism	9 (1.2%)	89 (1.9%)	
- Buddhism	0 (0.0%)	9(0.2%)	
- Others	11 (1.4%)	21 (0.5%)	
Marital status			* < 0.001
- Never married	189 (24.7%)	819 (17.7%)	
- Married	556 (72.7%)	3,646 (78.7%)	
- Divorced/Widowed	20 (2.6%)	167 (3.6%)	
Education level			* < 0.001
- Primary and under	8 (1.0%)	61 (1.3%)	
- Secondary	61 (8.0%)	704 (15.2%)	
- Higher	696 (91.0%)	3,867 (83.5%)	
Work type			* < 0.001
- No work	50 (6.5%)	289 (6.2%)	
- Public servants/army/police	539 (70.5%)	2,970 (64.1%)	
- Health workers	22 (2.9%)	390 (8.4%)	
- Entrepreneurs	22 (2.9%)	137 (3.0%)	
- Private sector	89 (11.6%)	582 (12.6%)	
- Farmer/fisherman/labor	6 (0.8%)	34 (0.7%)	
- Others	37 (4.8%)	230 (5.0%)	

Note: ^∗^*p* < 0.05

The study found that married respondents control both categories of information clarity about Covid-19 in Indonesia. Meanwhile, based on education level, respondents with higher education occupied both types of information clarity about Covid-19. Moreover, respondents with public servants/army/police ruled both categories of information clarity about Covid-19.

The subsequent analysis was a colinearity test of information clarity about Covid-19 in Indonesia. The test results revealed that there was no collinearity between independent variables. The tolerance value for all variables is more significant than 0.10; meanwhile, the variance inflation factor (VIF) value for all independent variables is less than 10.00. According to the evaluation results, there was no evidence of multicollinearity in the regression model.

Table 3 shows the binary logistic regression test result of information clarity about Covid-19 in Indonesia. The final test chose the "information clarity about Covid-19 = No" category as a reference.

**Table 3:** Binary logistic regression of information clarity about Covid-19 in Indonesia (*n* = 5,397)

Predictor	Information clarity about Covid-19			
	***p*-value**	**AOR**	**95% CI**	
			**Lower Bound**	**Upper Bound**
Newspaper/magazine: Every day	* < 0.001	1.670	1.327	2.101
Newspaper/magazine: 2–3 days a week	*0.011	1.386	1.078	1.783
Newspaper/magazine: 4–5 days a week	*0.027	1.470	1.045	2.067
Newspaper/magazine: > 5 days a week	-	-	-	-
Television: Every day	* < 0.001	1.601	1.307	1.962
Television: 2–3 days a week	*0.002	1.452	1.147	1.837
Television: 4–5 days a week	0.952	0.990	0.725	1.353
Television: > 5 days a week	-	-	-	-
Age group: 18–24	0.232	0.526	0.183	1.509
Age group: 25–34	*0.017	0.321	0.126	.813
Age group: 35–44	0.109	0.467	0.184	1.185
Age group: 45–54	0.977	0.986	0.384	2.535
Age group: 55–64	0.908	1.061	0.389	2.893
Age group: > 64	**-**	-	-	-
Religion: Moslem	*0.008	2.836	1.318	6.103
Religion: Christian	*0.013	2.776	1.239	6.220
Religion: Catholic	*0.040	2.499	1.044	5.981
Religion: Hinduism	*0.008	4.042	1.434	11.393
Religion: Buddhism	0.999	350,700,720.285	0.000	.-
Religion: Others	-	-	-	-
Marital status: Never married	-	-	-	-
Marital status: Married	0.859	0.981	0.797	1.208
Marital status: Divorced/Widowed	0.553	0.854	0.506	1.440
Education level: Primary and under	-	-	-	-
Education level: Secondary	0.510	1.314	0.584	2.955
Education level: Higher	0.180	0.578	0.260	1.288
Work type: No work	-	-	-	-
Work type: Public servants/army/police	* < 0.001	1.949	1.344	2.826
Work type: Health workers	* < 0.001	5.766	3.288	10.110
Work type: Entrepreneurs	0.208	1.438	0.817	2.531
Work type: Private sector	*0.003	1.869	1.243	2.809
Work type: Farmer/fisherman/labor	0.903	1.061	0.410	2.745
Work type: Others	*0.025	1.754	1.074	2.862

Note: ^∗^*p* < 0.05

Table 3 shows that people who read a newspaper/magazine every day are 1.670 times more likely than people who read a newspaper/magazine > 5 days a week to get clear information about Covid-19 in Indonesia (AOR 1.670; 95% CI 1.327–2.101). Meanwhile, people who read a newspaper/magazine 2–3 days a week are 1.386 times more likely than people who read a newspaper/magazine > 5 days a week to get precise information about Covid-19 in Indonesia (AOR 1.386; 95% CI 1.078–1.783). Moreover, people who read a newspaper/magazine 4–5 days a week are 1.470 times more likely than people who read a newspaper/magazine > 5 days a week to get precise information about Covid-19 in Indonesia (AOR 1.470; 95% CI 1.045–2.067). This analysis informs that frequency of reading a newspaper/magazine is one of the factors related to information clarity about Covid-19 in Indonesia.

Based on watching television, people watching television 2–3 days a week are 1.601 times more likely than people watching television > 5 days a week to get precise information about Covid-19 in Indonesia (AOR 1.601; 95% CI 1.307–1.962). Moreover, people watching television 4–5 days a week are 1.452 times more likely than people watching television > 5 days a week to get precise information about Covid-19 in Indonesia (AOR 0.452; 95% CI 1.147–1.837). This information shows that the frequency of watching television is partially related to information clarity about Covid-19 in Indonesia.

Apart from media exposure in the frequency of reading a newspaper/magazine and watching television, the analysis found three control variables related to information clarity about Covid-19 in Indonesia. The five variables are age, religion, and work type.

## Discussion

Newspaper/magazines and television have become frequently accessed by communities, especially in Indonesia. Both are dominating information exposure by millions of people in Indonesia. According to a survey by KIC, generally, Indonesian people seek information through social media 73%. For specific, online news placed to access 26,7% [22]. This matter has similarities to the people of Bangladesh. Most people clarify information, especially covid-19, using social media and television. Both were used to determine accurate and valid information [23].

The results indicate that people still choose visual media over audio or optical audio for clarity of information. Respondents prefer newspapers/magazines to other media because this is a medium where people can repeat the information they want and read it repeatedly for information clarity. On the other hand, the information displayed in newspapers/magazines is often accompanied by in-depth explanations so that it is more logically reliable [24, 25]. Information on the results of this study can lead health promoters, either in Indonesia or other countries similar to Indonesia, to present reliable information by conveying it in depth through newspapers/magazines or television.

Based on the result, the frequency of reading a newspaper/magazine to get information about covid-19 is the factor. The clarity is not away from the exposure of the media. The headline may help first; after all, the readers will follow the complete body paragraphs to find out the truth of the content. Usually, a piece of news in a newspaper/magazine will always be attracted by a visual and attractive headline. The condition applies an impression to the readers [26]. In contrast, the unfamiliar or unknown news content sometimes needs to be carefully read. They possibly open a piece of misleading information or hoaxes. The anonymous media may lead to uncertainty about covid-19 and confuse readers [27].

Less information to study about information clarity regarding the Covid-19 matter. In addition, how was the frequency of reading the newspaper and watching television to get information clarity? Moreover, how many of frequently do the audiences read or watch them? This study results in the information clarity about covid-19 is dominantly correlated to reading newspapers/magazines, and both are printed and digitalized (online).

Conversely, television is partially related to information clarity about Covid-19 in Indonesia. Television is an alternative medium for getting information clarity on covid-19. During the pandemic, the television volume to watch increased day by day [28]. The audiences in Indonesia are numerous, and almost 60% of people switch onto their television to access news or information [22]. It remains mainstream in Indonesia as people are directed to stay home and do their jobs, called work from home (WFH). It is the closest and easiest way to access information as you no more need an internet connection. Social distancing has also become protocol to limit the mobility of the communities. However, the bridge for social life focused on the television, the alternative way to clarify information. A study clearly stated that television is a social connection [29].

Controversies exactly appeared to the audiences of television in Indonesia. Sometimes, they blow up the booming headlines to attract them. The headlines then require the audiences to figure out whether or not covid-19 exists. The information is misleading and sometimes makes the government hardly clarify that news [30]. Moreover, the government may also point out the hoax producers. Misinformation about covid-19 happened in Bangladesh, and the government blamed initiating corruption during the pandemic [31]. The pandemic then is assumed to be how serious a country can overcome its problem and how they can ensure its people by valid and transparent information on covid-19.

However, television is closely related to government. The people require clarity to any information about covid-19. Evidence-based strategy and authorities of government to issue covid-19 clarity must be clear to the mind of their people [32]. Television has a great power to persuade people not to make safe from the covid-19 spreads. Exposing news by persuasion and strong arguments will relieve people and make them aware. The hoax news has to be addressed and countered. Hoaxes may lead the government into a serious problem. Moreover, clear information leads the audience to learn and follow instructions [12, 33].

Rogers stated that innovation diffusion first hit knowledge people usually have [14, 34]. Media play a crucial role in getting attention and giving the first impression of the audience's understanding. When cases of Covid-19 are diffused globally, the media rapidly publishes the news. The media bring the issues of Covid-19 to become so urgent that people have to be aware of this virus. Whether they like it or not, the knowledge of viruses will increase as long as the exposure to media increases [14].

The pandemic has become easily accessed by people around Indonesia, and the virus's risks and dangers are digitally displayed. People can protect and prevent themselves by watching and listening to mainstream or online media. They also read whenever and wherever they want to as the signal or internet connection is available. In Canada, television has become the media that watches more than other media [35, 36].

Nevertheless, information in the media probably leads to several opposed ideas or is sometimes fueled by a hoax [12]. This condition may lead to insecurity towards people who utilize the media. The government has to filter and regulate disinformation and fraud. They prepared an expert explanation to counter hoaxes [33]. Immunization may also be a part of the hoax counter, and it defeats the weak argument with the stronger one [30, 37]. The fake news disappears as long as the accurate information fights it. Health professionals and journalists may take over the misleading report [30]. The authorities must persuade real news against fake ones collaboratively [4].

The more people read, the more they believe. When someone experiences information and continually gets the same views, they may lead to a belief. The exposure of data, again and again, will make people exhausted, and then they trust it as absolute facts [5, 38]. The situation discussed earlier is related to the perceived information clarity of the community. However, the situation is still from the side that is accepted by the community, accuracy information still needs to be discussed further. The amount of hoax information also makes the situation worse [9, 11]. Such a dire situation may have resulted from celebrities' and influencers' perspectives on the COVID-19 pandemic conspiracy theory [12, 14]. This condition makes the government need to create a task force to address the situation [12].

Moreover, in addition to media exposure in the frequency of reading a newspaper/magazine and watching television. The five variables significantly correlated to information clarity about Covid-19 in Indonesia include age group, religion, and work type. This study confirms the results of previous studies conducted in various countries: India, Italy, Lebanon, and Vietnam [26, 39–42].

Religion strengthens a person's belief after he is clear with information, regardless of religion. Information clarity is also more reassuring, regardless of accuracy, but can lower their anxiety level [13, 43].

### Study limitation

This study limits the particular name of media accessed. The newspaper/magazine and television are specific to the program or media names, and this study cannot describe which one is printed or digitalized. The newspaper/magazine is seen as the same either offline or online. In addition, the television is merged whether watched via the internet or satellite-based. Therefore, when an audience accessed the internet and then enjoyed television accounts or platforms, they were acclaimed to use television when looking for information about covid-19. Moreover, this study only analyzes the frequency of media exposure, not the duration of media exposure.

## Conclusions

Based on the results, the study concluded that there were two types of media exposure related to information clarity about Covid-19 in Indonesia: the frequency of reading newspapers and watching television. On the other hand, the study found five control variables related to information clarity about Covid-19, i.e., age, religion, marital, education, and work type.

## Data Availability

The author cannot publicly share the data because a third party and the Ministry of Health of the Republic of Indonesia, who owns the data, do not have permission to share it. The 2018 Indonesian Basic Health Survey data set is available on the web http://www.litbang.kemkes.go.id/layanan-permintaan-data-riset/ for researchers who meet the criteria for access to confidential data.
